# Alpha‐Synuclein co‐pathology drives tau accumulation in Alzheimer's disease patients and iPSCs‐derived models

**DOI:** 10.1002/alz70855_106193

**Published:** 2025-12-24

**Authors:** Xiaoxuan Song, Teodoro De Vecchi, Jeannine Widmann, Federico Fierli, Viktoria Ruf, Qilin Tang, Thomas Arzberger, Sigrun Roeber, Thomas Köglsperger, Otto Windl, Christian Haass, Juliane Winkelmann, Jochen Herms, Felix Strübing

**Affiliations:** ^1^ Ludwig Maximilian University of Munich, Munich, Bavaria, Germany; ^2^ German Center for Neurodegenerative Diseases (DZNE), Munich, Bavaria, Germany; ^3^ LMU University Hospital, Munich, Bavaria, Germany; ^4^ Munich Cluster of Systems Neurology (SyNergy), Munich, Bavaria, Germany

## Abstract

**Background:**

The molecular basis for accelerated cognitive decline seen in Alzheimer's Disease (AD) cases presenting with cortical alpha‐Synuclein co‐pathology is not well understood. Mouse experiments have shown adverse interactions between tau (encoded by *MAPT*) and alpha‐Synuclein (encoded by *SNCA*), but how this finding translates to humans from a genome‐centered point of view remains unknown.

**Method:**

Whole genome sequencing was performed on 137 neuropathologically defined AD cases, 36 of which presented with neocortical alpha‐Synuclein co‐pathology (Braak stage 6). Polygenic risk scores were calculated. Single‐nucleus RNA sequencing and Western Blot data were collected from post‐mortem tissue. Transcriptomic and proteomic results were validated in the MSBB cohort (*n* >300). Cellular, molecular and epigenetic consequences were assessed in isogenic iPSCs‐derived neurons carrying a triplication of *SNCA* (AST) or a normal *SNCA* copy number (CAS).

**Result:**

AD brains with alpha‐Synuclein co‐pathology had significantly higher polygenic risk scores for Parkinson's Disease, which could be partially explained by variants associated with higher expression of *SNCA*. Single‐nucleus RNA sequencing and immunoblot analysis revealed a higher expression of *MAPT* and phosphorylated tau in alpha‐Synuclein co‐pathology cases. Protein and mRNA expression of *MAPT* and *SNCA* were positively correlated in the MSBB cohort. The employed iPSCs differentiation protocol accelerated neuronal maturation due to transient inhibition of *EZH2*. Day50 AST neurons exhibited significantly increased pathological tau and alpha‐Synuclein at both the RNA and protein levels compared to CAS neurons. AST neurons also showed highly activated GSK3β and decreased PSD95 (post‐synaptic protein) in the immunofluorescence and immunoblot analyses. ATAC profiles identified dysregulated accessibility in the cAMP signaling pathway, as well as pathways related to axon guidance, postsynaptic density, and calcium signaling, among others.

**Conclusion:**

We demonstrate that alpha‐Synuclein co‐pathology in AD is characterized by higher phosphorylated tau levels in patients and iPSC‐derived neurons. Our results provide insights into the complex molecular processes through which alpha‐Synuclein and tau synergistically drive dementia‐related pathology.

